# Benzamide-4-Sulfonamides Are Effective Human Carbonic Anhydrase I, II, VII, and IX Inhibitors

**DOI:** 10.3390/metabo8020037

**Published:** 2018-06-01

**Authors:** Morteza Abdoli, Murat Bozdag, Andrea Angeli, Claudiu T. Supuran

**Affiliations:** 1 Dipartimento Neurofarba, Sezione di Scienze Farmaceutiche e Nutraceutiche, Università degli Studi di Firenze, Via U. Schiff 6, Sesto Fiorentino, 50019 Florence, Italy; mortezaabdoli1987@gmail.com (M.A.); andrea.angeli@unifi.it (A.A.); 2 Department of Chemistry, Faculty of Science, Lorestan University, Khorramabad 6813833946, Iran

**Keywords:** carbonic anhydrase, human isoform, sulfonamide, benzamide, pathogens

## Abstract

A series of benzamides incorporating 4-sulfamoyl moieties were obtained by reacting 4-sulfamoyl benzoic acid with primary and secondary amines and amino acids. These sulfonamides were investigated as inhibitors of the metalloenzyme carbonic anhydrase (CA, EC 4.2.1.1). The human (h) isoforms hCA II, VII, and IX were inhibited in the low nanomolar or subnanomolar ranges, whereas hCA I was slightly less sensitive to inhibition (K_I_s of 5.3–334 nM). The β- and γ-class CAs from pathogenic bacteria and fungi, such as *Vibrio cholerae* and *Malassezia globosa*, were inhibited in the micromolar range by the sulfonamides reported in the paper. The benzamide-4-sulfonamides are a promising class of highly effective CA inhibitors.

## 1. Introduction

**Figure 1:**
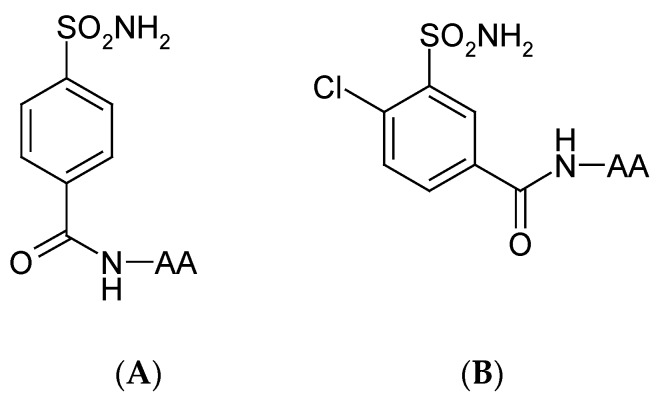
(**A**,**B**) Sulfonamides incorporating benzamide moieties, amino acid (AA) and dipeptide AA moieties [1,2].

Benzamides incorporating 3- or 4-sulfamoyl moieties, such as derivatives **A** and **B** (Figure 1) were investigated [1,2] as inhibitors of the zinc metallo-enzyme carbonic anhydrase (CA, EC 4.2.1.1) [3,4,5,6,7,8,9,10,11,12] in this study, in the search of agents with intraocular pressure lowering effects [1,2]. The incorporation of a wide range of amino acid (AA) or dipeptide AA moieties in molecules **A** and **B** led to enhanced water solubility for topical administration within the eye. These compounds showed remarkable in vitro inhibitory effects, assayed by an esterase method with 4-nitrophenyl acetate as substrate, against isoforms hCA II and IV, involved in aqueous humor production within the eye [1,2,3,4,5,6,7,8,9,10,11,12].

The CA inhibitors (CAIs) belonging to the sulfonamide and sulfamate types have been used clinically for several decades as diuretics [13,14], antiglaucoma agents [15], and anti-obesity drugs [16,17]. More recently, a large number of studies showed that CA inhibition has profound antitumor effects by inhibiting hypoxia-inducible isoforms hCA IX and XII, overexpressed in many hypoxic tumors [18,19,20,21,22]. Furthermore, several proof-of-concept studies demonstrated the involvement of some CA isoforms in neuropathic pain [23,24] and arthritis [25,26], with the CAIs of sulfonamide and coumarin [27,28,29,30] types demonstrating significant in vivo effects in animal models of these diseases. Thus, the field of drug design, synthesis, and in vivo investigations of various types of CAIs is highly dynamic, with the action of a large number of interesting new chemotypes on these widespread enzymes being constantly studied [27,28,29,30,31,32,33,34,35,36,37,38,39]. As they catalyze the interconversion between carbon dioxide (CO_2_) and bicarbonate with the formation of a proton, CAs are widespread in organisms all over the phylogenetic tree as seven distinct genetic families: the α-, β-, γ-, δ-, η-, ξ-, and θ-CAs [3,4,5,6,7,8,9,10,11,12,40,41,42,43,44,45,46,47]. CAs participate in crucial physiologic processes connected to pH homeostasis, metabolism, transport of gases and ions, and secretion of electrolytes in virtually all living beings [3,4,5,6,7,8,9,10,11,12,40,41,42,43,44,45,46,47].

Apart from the inhibition of human (h) or other vertebrate CA isoforms, the interest in inhibiting such enzymes present in various pathogenic organisms (bacteria, fungi, protozoa, or worms) has presented the possibility of designing anti-infective agents with a novel mechanism of action [40,41,42,43,44,45,46,47,48,49,50,51]. Thus, in this paper, we explored novel CAIs belonging to the sulfonamide class, incorporating benzamide moieties similar to compounds reported earlier, but that were investigated for the inhibition of isoforms involved in important diseases, such as glaucoma (hCA II), neuropathic pain (hCA VII), or tumors (hCA IX), and ubiquitous off target isoform hCA I. Furthermore, we investigated whether this chemotype shows inhibitory effects against β- and γ-class CAs from pathogenic bacteria (*Vibrio cholerae*) or fungi (*Malassezia globosa*).

## 2. Results

### 2.1. Chemistry

**Scheme 1:**
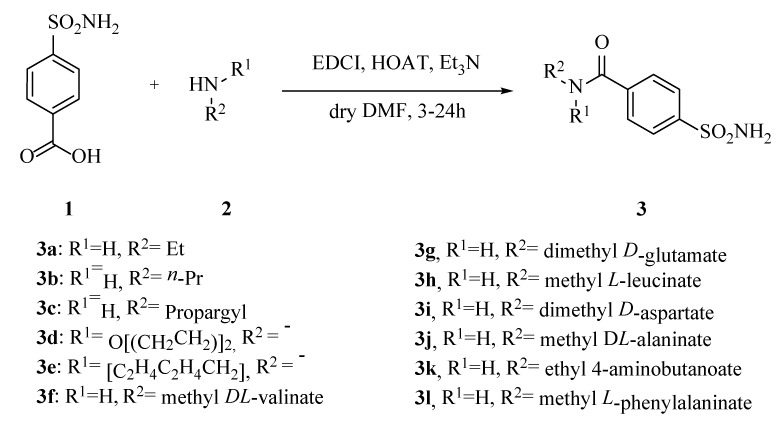
Synthesis of compounds **3a**–**l**.

The classical coupling of carboxylic acid **1** with amines, in the presence of carbodiimides (EDCI) and hydroxybenzotriazole has been used for synthesis, as reported previously [1,2] (Scheme 1).

Compound **1** was condensed with compounds **3a**–**e** that possess primary or secondary amines as well amino acid derivatives **3f**–**l** in the presence of EDCI and 1-hydroxy-7-azabenzotriazole (HOAT) to obtain their corresponding amides (Scheme 1). By choosing variously substituted amines and amino acids, incorporating both simple aliphatic and heterocyclic scaffolds (for the amine) and aliphatic and aromatic amino acids, the physico-chemical properties and enzyme inhibitory properties of the new compounds could be modulated. For example, the amino acid derivatives **3f**, **3g**, **3h**, **3j**, and **3l** may form sodium salts leading to water soluble CAIs.

### 2.2. Carbonic Anhydrase Inhibition

Sulfonamides **3a**–**3l** were tested as inhibitors of four hCAs involved in various pathologies, hCA I, II, VII, and IX, as well as three β- and γ-CAs from pathogenic organisms: the β-CAs from the bacterium *Vibrio cholerae* (VchCAβ) and the fungus *Malassezia globosa* (MgCA), and the γ-CA from the same pathogenic bacterium, VchCAγ–enzymes recently cloned and characterized by our group as potential anti-infective targets [52,53,54,55,56,57,58,59] (Table 1).

## 3. Discussion

The following structure-activity relationship (SAR) were determined from the data of Table 1, in which the standard sulfonamide inhibitor acetazolamide (**AAZ**) was also included for comparison.

The slow cytosolic isoform hCAI, involved in some ocular diseases (not glaucoma) [3,4,5,6,7], was inhibited by sulfonamides **3a**–**l** reported here with K_I_s in the range of 5.3 to 334 nM. The ethyl- (**3a**) derivative was the weakest inhibitor, whereas **3c**, **3f**, **3i**, and **3j** showed medium potency inhibitory action, with a K_I_s in the range of 57.8 to 85.3. These compounds incorporate propargyl, valyl, aspartyl, and alanyl moieties. The remaining derivatives, **3b**, **3d**, **3e**, **3g**, **3h**, **3k**, and **3l** showed very effective hCA I inhibitory properties, with a K_I_s in the range of 5.3 to 29.7 nM, being CAIs an order of magnitude better compared to acetazolamide (Table 1). Small changes in the scaffold (compare **3a** and **3b**) led to dramatic changes in the hCA I inhibitory effects, with the propyl derivative **3b** being 40.7 times more effective an inhibitor compared with the ethyl derivative **3a**.

All sulfonamides **3a**–**l** reported here were excellent hCA II inhibitors, with a K_I_s in the range of 1.9 to 7.0 nM, thus being more effective than **AAZ** (Table 1). With this highly effective inhibition and small range in the variation of the K_I_s, the SAR is flat and the only conclusion is that all the explored substitution patterns led to highly effective hCA II inhibitors. This is also the dominant cytosolic isoform, involved in glaucoma, diuresis, respiration, and electrolyte secretion in a multitude of tissues [3,4,5,6,7,8,9,10,11,12], meaning these results are highly significant.

The third cytosolic isoform investigated here, hCA VII, predominantly found in the brain and involved in epileptogenesis and neuropathic pain [16,17,18,19,20,21,22,23,24], was also effectively inhibited by sulfonamides **3a**–**l**, which showed a K_I_s in the range of 0.4 to 26.7 nM. Most of these compounds were sub-nanomolar hCA VII inhibitors (e.g., **3b**–**3d, 3g**–**3i, 3k, 3l**), being more effective by an order of magnitude compared with the standard **AAZ**, whereas few of them showed the same potency as **AAZ** (**3e**, **3f**, **3j**) and only the ethyl derivative **3a** was a less effective inhibitor compared to **AAZ**, with a K_I_ of 26.7 nM. Overall, the SAR is extremely simple, and except for the ethyl derivative mentioned above, all the substitution patterns from derivatives **3b**–**3l** indicated all compounds are highly effective hCA VII inhibitors.

The tumor-associated, hypoxia-inducible isoform hCA IX was effectively inhibited by sulfonamides **3a**–**l**, with a K_I_s in the range of 8.0 to 26. 0 nMh. **AAZ** has an inhibition constant of 25.8 nM against this isoform. The most effective inhibitors, **3h** and **3k**, with a K_I_s of 8.0–9.3 nM, incorporated amino acyl moieties, but all substitution patterns present in compound **3**, of the amine or amino acid type, led to highly effective hCA IX inhibition.

Conversely, the β- and γ-CAs from pathogenic organisms investigated here were poorly inhibited by these compounds, which showed activity in the micromolar range, with few exceptions (Table 1). Thus, for VchCAβ, the K_I_s was in the range of 0.41 to 8.58 µM; for MgCA, in the range of 87.3 nM to 7.67 µM; and for VchCAγ, in the range of 0.27 to 4.45 µM. Notably, **3j** compounds, which incorporate the alanyl moiety, showed a good inhibitory effect against the *Malassezia* enzyme, one of the causative agents of dandruff. Acetazolamide is a highly ineffective MgCA inhibitor, and most other sulfonamides investigated here, although less effective than **3j**, showed a better activity compared with the standard sulfonamide CAI. Overall, β- and γ-CAs are less sensitive to inhibition with sulfonamides compared with α-CAs [3,4,5,6,7,8,9,10,11,12,13,14].

## 4. Materials and Methods

### 4.1. Chemistry

Amines, 4-sulfamoyl-benzoic acid, buffers, solvents, and acetazolamide (**AAZ**) were commercially available, obtained as highest purity reagents from Sigma-Aldrich/Merck, Milan, Italy. Nuclear magnetic resonance (^1^H NMR, ^13^C NMR) spectra were recorded using a Bruker Avance III 400 MHz spectrometer (Bruker, Billerica, MA, USA) in dimethyl sulfoxide (DMSO-*d_6I_*). Chemical shifts are reported in parts per million (ppm) and the coupling constants (*J*) are expressed in Hertz (Hz). Splitting patterns were designated as follows: s, singlet; d, doublet; t, triplet; m, multiplet; brs, broad singlet; and dd, double of doubles. The assignment of exchangeable protons (OH and NH) was confirmed by the addition of D_2_O. Analytical thin-layer chromatography (TLC) was performed on Merck silica gel F-254 plates. Flash chromatography purifications were performed on Merck Silica gel 60 (230–400 mesh ASTM) as the stationary phase and MeOH/DCM were used as eluents.

#### 4.1.1. General Procedure to Synthesize Compounds **3a**–**l**

A solution of 4-carboxybenzene sulfonamide **1** (1.0 eq) in dry dimethylformamide (DMF, 3–5 mL) was treated with primary or secondary amines or amino acids **2a**–**l** (1.2 eq), then followed by addition of *N*-(3-dimethylaminopropyl)-*N*′-ethylcarbodiimide hydrochloride (EDCI, 1.5 eq.), 1-hydroxy-7-azabenzotriazole (HOAT, 1.5 eq), and triethylamine (Et_3_N, 3 eq). The reaction continued until the consumption of starting materials (TLC monitoring, 3–24 h) and quenched with water. The title compounds were either obtained from filtration of the precipitates formed followed by washing with water (**3a**–**3e**, **3h, 3k**–**l**) or extracted from ethyl acetate (EtOAc). In the latter, the combined organic layers were washed with H_2_O (3 × 20 mL), dried over sodium sulfate, filtered, and concentrated in a vacuum to provide a residue that was triturated from dichloromethane (**3f**–**g**, **3i**–**j**).

#### 4.1.2. Characterization of Synthesized Compounds (**3a**–**l**)

*N*-Ethyl-4-Sulfamoylbenzamide (**3a**): 140 mg white solid, yield 83%; δ_H_ (400 MHz, DMSO-*d*_6_) 1.17 (3H, t, *J* 7.2), 3.33 (2H, m), 7.51 (2H, s, exchange with D_2_O, SO_2_NH_2_), 7.93 (2H, d, *J* 8.4), 8.02 (2H, d, *J* 8.4), 8.68 (1H, t, *J* 7.2 exchange with D_2_O, NH); δ_C_ (100 MHz, DMSO-*d*_6_) 15.5, 35.1, 126.5, 128.6, 138.5, 147.0, and 165.8; *m/z* (ESI positive) 229.0 [M + H]^+^.

*N*-Propyl-4-Sulfamoylbenzamide (**3b**): 120 mg white solid, yield 80%; δ_H_ (400 MHz, DMSO-*d*_6_) 0.93 (3H, t, *J* 7.2), 1.58 (2H, m), 3.27 (2H, q, *J* 7.2), 7.50 (2H, s, exchange with D_2_O, SO_2_NH_2_), 7.93 (2H, d, *J* 8.4), 8.02 (2H, d, *J* 8.4), 8.66 (1H, t, *J* 7.2, exchange with D_2_O, NH); δ_C_ (100 MHz, DMSO-*d*_6_) 12.3, 23.2, 42.0, 126.5, 128.7, 138.5, 147.0, 166.0; *m/z* (ESI positive) 243.1 [M + H]^+^.

*N*-(Prop-2-Yn-1-Yl)-4-Sulfamoylbenzamide (**3c**): 120 mg yellow solid, yield 74%; δ_H_ (400 Mhz, DMSO-*d*_6_) 3.18 (1H, T, *J* 2.5), 4.12 (2H, dd, *J* 5.5, 2.5), 7.52 (2H, s, exchange with D_2_O, SO_2_NH_2_), 7.94 (2H, d, *J* 8.8), 8.04 (2H, d, *J* 8.8), 9.16 (1H, t, *J* 5.5, exchange with D_2_O, N*H*); δ_c_ (100 Mhz, DMSO-*d*_6_) 29.5, 73.9, 81.9, 126.5, 128.8, 137.6, 147.3, 165.8; *m/z* (ESI Positive) 239.0 [M + H]^+^. Experimental data in agreement with reported data [61].

4-(Morpholine-4-Carbonyl)Benzenesulfonamide (**3d**): 10 mg pale yellow solid; 9% yield; δ_H_ (400 MHz, DMSO-*d*_6_) 3.65 (8H, m), 7.44 (2H, s, exchange with D_2_O, SO_2_NH_2_), 7.63 (2H, d, *J* 8.0), 7.92 (2H, d, *J* 8.0); δ_C_ (100 MHz, DMSO-*d*_6_) 66.9, 66.9, 126.7, 128.5, 139.7, 145.8, 168.8; *m/z* (ESI positive) 271.1 [M + H]^+^.

4-(Piperidine-1-Carbonyl)Benzenesulfonamide (**3e**): 12 mg yellow solid, yield 18%; δ_H_ (400 MHz, DMSO-*d*_6_) 1.50 (2H, m), 1.65 (4H, m), 3.25 (2H, m), 3.63 (2H, m), 7.48 (2H, s, exchange with D_2_O, SO_2_NH_2_), 7.59 (2H, d, *J* 8.0), 7.91 (2H, d, *J* 8.0); δ_C_ (100 MHz, DMSO-*d*_6_) 26.1, 26.7, 48.8, 126.7, 128.0, 140.7, 145.4, 168.5; *m/z* (ESI positive) 269.1 [M + H]^+^.

Methyl (4-Sulfamoylbenzoyl)-dl-Valinate (**3f**): 12 mg pale yellow solid, yield 10%; δ_H_ (400 MHz, DMSO-*d*_6_) 0.98 (3H, d, *J* 6.8), 1.02 (3H, d, *J* 6.8), 2.23 (1H, m), 3.70 (3H, s), 4.36 (1H, t, *J* 6.8), 7.53 (2H, s, exchange with D_2_O, SO_2_NH_2_), 7.94 (2H, d, *J* 8.4), 8.05 (2H, d, *J* 8.4), 8.83 (1H, d, *J* 6.8, exchange with D_2_O, NH); δ_C_ (100 MHz, DMSO-*d*_6_) 19.9, 20.0, 30.5, 52.6, 59.6, 126.4, 129.2, 137.7, 147.4, 167.0, 172.9; *m/z* (ESI positive) 315.0 [M + H]^+^.

Dimethyl (4-Sulfamoylbenzoyl)-d-Glutamate (**3g**): 14 mg pale yellow solid, yield 16%; δ_H_ (400 Mhz, DMSO-*d*_6_) 2.07 (2H, m), 2.16 (2H, m), 3.63 (3H, s), 3.70 (3H, s), 4.53 (1H, m), 7.52 (2H, s, exchange with D_2_O, SO_2_NH_2_), 7.94 (2H, d, *J* 8.8), 8.05 (2H, d, *J* 8.8), 8.83 (1H, d, *J* 7.3, exchange with D_2_O, NH); δ_C_ (100 Mhz, DMSO-*d*_6_) 26.6, 30.8, 52.3, 52.9, 53.0, 126.5, 129.0, 137.4, 147.5, 166.6, 172.9, 173.5; *m/z* (ESI Positive) 359.1 [M + H]^+^.

Methyl (4-Sulfamoylbenzoyl)-l-Leucinate (**3h**): 37 mg white solid, yield 25%; δ_H_ (400 Mhz, DMSO-*d*_6_) 0.92 (3H, d, *J* 6.4), 0.97 (3H, d, *J* 6.4), 1.63 (1H, m), 1.70–1.86 (2H, m), 3.69 (3H, s), 4.56 (1H, m), 7.52 (2H, s, exchange with D_2_O, SO_2_NH_2_), 7.95 (2H, d, *J* 8.3), 8.06 (2H, d, *J* 8.3), 8.94 (1H, d, *J* 6.4, exchange with D_2_O, NH); δ_C_ (100 Mhz, DMSO-*d*_6_) 22.1, 23.7, 25.3, 40.2, 51.9, 52.8, 126.5, 129.0, 137.5, 147.4, 166.5, 173.8; *m/z* (ESI Positive) 329.01 [M + H]^+^.

Dimethyl (4-Sulfamoylbenzoyl)-d-Aspartate (**3i**): 35 mg yellow solid, yield 50%; δ_H_ (400 Mhz, DMSO-*d*_6_) 2.86–3.04 (2H, m), 3.67 (3H, s), 3.70 (3H, s), 4.89 (1H, m), 7.52 (2H, s, exchange with D_2_O, SO_2_NH_2_), 7.96 (2H, d, *J* 8.7), 8.03 (2H, d, *J* 8.7), 9.13 (1H, d, *J* 7.6, exchange with D_2_O, NH); δ_C_ (100 Mhz, DMSO-*d*_6_) 36.2, 50.2, 52.6, 53.2, 126.6, 129.0, 137.2, 147.6, 166.1, 171.3, 171.9; *m/z* (ESI Positive) 345.0 [M + H]^+^.

Methyl (4-Sulfamoylbenzoyl)-dl-Alaninate (**3j**): 14 mg white solid, yield 13%; δ_H_ (400 Mhz, DMSO-*d*_6_) 1.46 (3H, d, *J* 7.3), 3,69 (3H, s), 4.54 (1H, m), 7.51 (2H, s, exchange with D_2_O, SO_2_NH_2_), 7.96 (2H, d, *J* 8.4), 8.06 (2H, d, *J* 8.4), 9.01 (1H, d, *J* 7.3, exchange with D_2_O, NH); δ_C_ (100 Mhz, DMSO-*d*_6_) 17.6, 49.3, 52.8, 126.5, 129.0, 137.4, 147.4, 166.2, 173.8; *m/z* (ESI Positive) 287.0 [M + H]^+^.

Ethyl 4-(4-Sulfamoylbenzamido)Butanoate *(***3k**): 80 mg white solid, yield 58%; δ_H_ (400 Mhz, DMSO-*d*_6_) 1.20 (3H, t, *J* 7.2), 1.83 (2H, pent, *J* 6.8), 2.39 (2H, t, *J* 6.8), 3.31 (2H, m), 4.09 (2H, q, *J* 7.2), 7.47 (2H, s, exchange with D_2_O, SO_2_NH_2_), 7.91 (2H, d, *J* 8.0), 8.01 (2H, d, *J* 8.0), 8.66 (1H, t, *J* 6.8, exchange with D_2_O, NH); δ_C_ (100 Mhz, DMSO-*d*_6_) 15.0, 25.3, 31.9, 39.6, 60.6, 126.5, 128.7, 138.3, 147.1, 166.1, 173.5; *m/z* (ESI Positive) 315.0 [M + H]^+^.

Methyl (4-Sulfamoylbenzoyl)-l-Phenylalaninate (**3l**): 130 mg white solid, yield 72%; δ_H_ (400 Mhz, DMSO-*d*_6_) 3.10–3.25 (2H, m), 3.69 (3H, s), 4.70–4.76 (1H, m), 7.24 (1H, m), 7.32 (4H, m), 7.52 (2H, s, exchange with D_2_O, SO_2_NH_2_), 7.93 (2H, d, *J* 8.4), 7.98 (2H, d, *J* 8.4), 9.08 (1H, d, *J* 7.9, exchange with D_2_O, NH); δ_C_ (100 Mhz, DMSO-*d_6_*) 37.1, 52.9, 55.2, 126.5, 127.4, 128.9, 129.2, 130.0, 137.4, 138.4, 147.4, 166.3, 172.8; *m/z* (ESI Positive) 363.0 [M + H]^+^.

### 4.2. CA Enzyme Inhibition Assay

An Sx.18Mv-R Applied Photophysics (Oxford, U.K.) stopped-flow instrument was used to assay he catalytic activity of various CA isozymes for CO_2_ hydration reaction [60]. Phenol red, at a concentration of 0.2 mM, was used as an indicator, working at the absorbance maximum of 557 nm, with 10 mM Hepes (pH 7.5, for α-CAs) or TRIS (pH 8.3, for β- and γ-CAs) as buffers, 0.1 M sodium sulfate (Na_2_SO_4_) (for maintaining constant ionic strength), following the CA-catalyzed CO_2_ hydration reaction for a period of 10 s at 25 °C. The CO_2_ concentrations ranged from 1.7 to 17 mM for the determination of the kinetic parameters and inhibition constants. For each inhibitor, at least six traces of the initial 5–10% of the reaction were used for determining the initial velocity. The uncatalyzed rates were determined in the same manner and subtracted from the total observed rates. Stock solutions of inhibitors (10 mM) were prepared in distilled-deionized water. Dilutions up to 1 nM were performed thereafter with the assay buffer. Enzyme and inhibitor solutions were pre-incubated together for 15 min (standard assay at room temperature) prior to assay, to allow for the formation of the enzyme–inhibitor complex. The inhibition constants were obtained by non-linear least-squares methods using PRISM 3 and the Cheng-Prusoff equation, as reported earlier [62,63,64,65,66,67,68,69,70,71,72,73,74,75]. All CAs were recombinant proteins produced as reported earlier by our groups [52,53,54,55,56,57,58,59,60,61,62,63,64,65,66,67,68,69,70,71,72,73,74,75,76].

## 5. Conclusions

We report a series of benzamides incorporating 4-sulfamoyl moieties, which were obtained by reacting 4-sulfamoyl benzoic acid with primary and secondary amines and amino acids. These sulfonamides were investigated as inhibitors of several enzymes, including the human (h) isoforms hCA II, VII, and IX, involved in severe pathologies, such as glaucoma, epilepsy, neuropathic pain and cancer; and β- and γ-class CAs from pathogenic bacteria and fungi. hCA II, VII, and IX were inhibited in the low nanomolar or subnanomolar ranges by all investigated sulfonamides, whereas hCA I was slightly less sensitive to inhibition (K_I_s of 5.3–334 nM). The *Vibrio cholerae* and *Malassezia globosa* CAs were generally inhibited in the micromolar range by the sulfonamides reported in the paper. The benzamide-4-sulfonamides constitute a promising class of highly effective CA inhibitors. Further investigations will focus on extending the series of sulfanilamide possessing aliphatic tails with carbamide linkers, such as cyclic and aliphatic and aromatic, to investigate and obtain isoform selective inhibitors for their profiling and possible in vivo applications.

## Figures and Tables

**Table 1:** Inhibition data of human carbonic anhydrase (CA) isoforms hCA I, II, VII, IX, and pathogenic bacteria and fungi β- and γ-CAs with compounds **3a**–**3l** in comparison with the standard sulfonamide inhibitor **AAZ** by a stopped flow carbon dioxide (CO_2_) hydrase assay [60].

	K_I_ (nM) ^a^						
Cpd	hCA I	hCA II	hCA VII	hCA IX	VchCAβ	MgCA	VchCAγ
**3a**	334	5.3	26.7	15.9	7082	7669	929
**3b**	8.2	3.5	0.4	26.0	7680	3921	636
**3c**	67.6	1.9	0.6	22.9	741	5781	383
**3d**	8.7	6.2	0.8	10.7	8587	5880	693
**3e**	29.7	7.0	6.2	18.1	749	3985	453
**3f**	57.8	4.5	3.7	16.0	8172	5500	4458
**3g**	8.2	5.2	0.6	19.7	862	632	503
**3h**	5.6	3.7	0.4	8.0	719	763	891
**3i**	75.7	6.1	0.7	12.1	910	6946	744
**3j**	85.3	6.1	3.7	21.5	412	87.3	271
**3k**	5.3	4.0	0.4	9.3	953	6695	756
**3l**	5.6	3.3	0.5	19.2	663	517	409
**AAZ**	250.0	12.1	5.7	25.8	451	74000	473

^a^ Mean from three different assay using a stopped flow technique. Errors were in the range of ±5% to 10% of the reported values.
